# Can acetylcysteine ameliorate cisplatin‐induced toxicities and oxidative stress without decreasing antitumor efficacy? A randomized, double‐blind, placebo‐controlled trial involving patients with head and neck cancer

**DOI:** 10.1002/cam4.2072

**Published:** 2019-04-11

**Authors:** Marília B. Visacri, Júlia C. F. Quintanilha, Vanessa M. de Sousa, Laís S. Amaral, Rosiane de F. L. Ambrósio, Luciane Calonga, Silvia F. B. B. Curi, Mayra F. de T. Leme, Carlos T. Chone, João M. C. Altemani, Priscila G. Mazzola, Carina Malaguti, Aníbal E. Vercesi, Carmen S. P. Lima, Patricia Moriel

**Affiliations:** ^1^ School of Medical Sciences University of Campinas Campinas Brazil; ^2^ Faculty of Pharmaceutical Sciences University of Campinas Campinas Brazil; ^3^ Clinical Hospital University of Campinas Campinas Brazil

**Keywords:** acetylcysteine, cisplatin, head and neck neoplasms, oxidative stress, toxicities

## Abstract

The protective antioxidant activity of acetylcysteine (NAC) against toxicity due to cisplatin has been reported in experimental models; however, its efficacy in patients has not been elucidated. The aim of this study was to investigate the possible protective effect of NAC on cisplatin‐induced toxicity and the effect of NAC on clinical response and oxidative stress in patients treated for head and neck cancer. This was a randomized, double‐blind, placebo‐controlled trial conducted in patients receiving high‐dose cisplatin chemotherapy concomitant to radiotherapy. Patients were randomly assigned to groups and received: (a) 600 mg NAC syrup, orally once daily at night for 7 consecutive days or (b) placebo, administered similarly to NAC. Nephro‐, oto‐, hepato‐, myelo‐, and gastrointestinal toxicities, clinical responses, and plasma and cellular markers of oxidative stress were evaluated. Fifty‐seven patients were included (n = 28, NAC arm; and n = 29, placebo arm). A high prevalence of most types of toxicities was observed after cisplatin chemotherapy; however, the parameters were similar between the two groups. There was a predominance of partial response to treatment. In the cellular and plasmatic oxidative stress analyses, minor differences were observed. Overall, there was no statistically significant difference between the groups for all outcomes. These findings show that low‐dose oral NAC does not protect patients with head and neck cancer from cisplatin‐induced toxicities and oxidative stress. The antitumor efficacy of cisplatin was apparently not impaired by NAC.

## 1 INTRODUCTION

Cisplatin is an anticancer drug most effective for the treatment of solid tumors, such as head and neck cancer. However, cisplatin causes nephrotoxicity, ototoxicity, hepatotoxicity, myelosuppression, and nausea and vomiting, which limit its use. Our previous studies showed that patients experienced anemia, lymphopenia, and nausea; had significant changes in serum creatinine and creatinine clearance; and experienced a reduction in the quality of life following treatment with cisplatin.^1^, ^2^

Oxidative stress is the main mechanism responsible for cisplatin‐induced toxicity. It is known that cisplatin leads to the generation of mitochondrial reactive oxygen species (ROS).^3^, ^4^ In addition, cisplatin binds to glutathione by glutathione‐S‐transferase to be eliminated from the cell, thus depleting cellular glutathione content.^5^ Consequently, after cisplatin treatment, oxidative stress biomarkers increase in the plasma and urine and antioxidants decrease.^6^, ^7^, ^8^, ^9^, ^10^ Based on this, several antioxidant compounds have been studied as possible protective agents against toxicities caused by cisplatin.^6^, ^7^

Acetylcysteine (NAC) is a drug used in clinical practices as a mucolytic agent, as an antidote for paracetamol poisoning, and as a protector of contrast‐induced nephropathy. It possesses antioxidant properties and is also a glutathione precursor.^14^ NAC has been studied in the context of cisplatin‐induced toxicities, and has been shown to protect against nephrotoxicity,^14^ ototoxicity,^15^ and hepatotoxicity^16^ in animal models and in vitro; however, its efficacy in patients has not been elucidated.

In terms of the effect of antioxidants on the clinical responses to cisplatin, the findings of one study suggested that antioxidants may decrease cisplatin's antitumor efficacy.^17^ Contrarily, an animal model study showed that the administration of NAC 4 h after cisplatin administration did not affect the antitumor action of cisplatin, in contrast to when NAC was administered 30 min earlier.^18^

The aim of this study was to investigate the possible protective effect of NAC on cisplatin‐induced toxicities and the effect of NAC on the clinical responses and oxidative stress in patients treated with cisplatin for head and neck cancer. This is the first randomized, double‐blind, placebo‐controlled trial to investigate this issue.

## 2 METHODS

### 2.1 Study design

This was a randomized, double‐blind, placebo‐controlled trial conducted from March 2015 to February 2017 at the clinical oncology department of a teaching hospital in São Paulo, Brazil. The Research Ethics Committee of the institution approved this study (number 30216814.9.0000.5404), and all patients signed a consent form authorizing the use of their data. The study was registered in ClinicalTrials.gov (NAC + Cisplatin2014, NCT 02241876).

### 2.2 Eligibility criteria

The study included outpatients with head and neck squamous cell carcinomas (oral cavity, oropharynx, hypopharynx, and larynx), who had not undergone any previous oncological treatment (surgery, chemotherapy, or radiotherapy) and were on high‐dose cisplatin chemotherapy with concurrent radiotherapy.

Patients were excluded if: distant metastasis or a second primary tumor was present; they declined to participate at any time during the course of the study; died before the start of the treatment; changed their chemotherapy protocol before the start of the treatment (cisplatin had been prescribed; however, for some reason, cisplatin was contraindicated and the prescription changed); did not tolerate the use of syrup or were administered the syrup <70% of the time; or were receiving nephrotoxic and ototoxic drugs during the treatment.

### 2.3 Cancer treatment protocol

Chemotherapy comprised three cycles of high‐dose (80‐100 mg/m^2^) cisplatin administered on days 1, 22, and 43. On each day of chemotherapy, the patients received vigorous hydration (3 L of saline solution 0.9%), diuretics (125 mL of mannitol 20%), electrolytes (20 mL of potassium chloride 19.1% and 10 mL of magnesium sulfate 10%), and prophylaxis of acute emesis (20 mg of dexamethasone plus 24 mg of ondansetron).

Concomitant with the three cycles of chemotherapy, the patients received 70 Gy of radiation therapy divided into 35 daily applications of 2 Gy administered for 5 days per week for 7 weeks. Radiation was performed using cobalt‐60 (Alcyon II, GE, France) and a linear accelerator (6 MV) (Varian Medical Systems, CA).

### 2.4 NAC and placebo arms

Patients included in the study were randomized into an NAC arm or a placebo arm through a randomization system (http://www.randomizer.org/). Both the researchers and the patients were blinded to this process during the study.

Patients in the NAC arm received 600 mg of NAC syrup (EMS^®^) administered orally once daily at night for 7 consecutive days (2 days before chemotherapy, on the day of chemotherapy, and 4 days after chemotherapy) in each cycle of chemotherapy. Those in the placebo arm received syrup without the NAC ingredient with the same schedule as the NAC arm. The excipients of both syrups were: propylene glycol, sodium hydroxide, sodium cyclamate, sodium saccharin, methylparaben, hyetellose, disodium EDTA, raspberry essence, propylparaben, and purified water. A self‐reported compliance form was used to monitor adherence to syrup consumption.

### 2.5 Demographic and clinical data

Data regarding patient characteristics were obtained from medical records and interviews with patients, including information concerning age, gender, race, comorbidities, Karnofsky Performance Status (KPS),^19^ smoking and drinking categories based on the studies by Jindal et al^20^ and Whitcomb et al,^21^ tumor differentiation, and the site and TNM stage of tumors.^22^

### 2.6 Toxicity of treatment

Each patient had their blood collected prior to cisplatin administration, and 5 and 20 days after each treatment cycle. Thus, nephrotoxicity (increased serum creatinine, decreased creatinine clearance estimated using the Cockcroft—Gault formula, hyperuricemia, hyponatremia, hypomagnesemia, hypokalemia, hypophosphatemia, and hypocalcemia), hepatotoxicity (hypoalbuminemia, increased serum level of aspartate aminotransferase, alanine aminotransferase, alkaline phosphatase, and total bilirubin), and myelotoxicity (anemia, leukopenia, neutropenia, lymphopenia, and thrombocytopenia) were investigated. Gastrointestinal toxicity (nausea and vomiting) was also studied considering the symptoms on day 1 (first 24 h after chemotherapy—acute phase) through day 5 (24‐120 h after chemotherapy—delayed phase). Furthermore, audiometric tests were performed before and 20 days after the end of cisplatin treatment to analyze ototoxicity. All toxicities were graded according to National Cancer Institute Common Toxicity Criteria for Adverse Events version 4.^23^

### 2.7 Clinical response

Before starting treatment and 30 days after the end of the treatment, the patients underwent computed tomography or magnetic resonance imaging. Clinical responses were classified into complete response, partial response, progressive disease, and stable disease according to the Response Evaluation Criteria in Solid Tumors 1.1 (RECIST 1.1).^24^

### 2.8 Cellular and plasmatic oxidative stress

Blood was collected prior to cisplatin administration, and at 5 and 20 days after initiating therapy. The blood was collected in vacuum tubes containing EDTA or citrate as the anticoagulant. For the cellular oxidative stress analysis, peripheral blood mononuclear cells (PBMCs) were isolated using HISTOPAQUE‐1077 (Sigma‐Aldrich, St. Louis, MO). Mitochondrial superoxide anion (O_2_^•‐^) and cellular H_2_O_2_ production were measured using MitoSOX^TM^Red and Amplex^®^ Red reagent, respectively, according to Quintanilha et al^25^. Other oxidative stress biomarkers, including free 8‐isoprostane, malondialdehyde (MDA), and total antioxidant capacity (TAC), were measured in the plasma obtained by centrifugation of whole blood. The levels of 8‐isoprostane were determined by enzyme‐linked immunosorbent assay (8‐Isoprostane EIA Kit; Cayman Chemical Company, USA). MDA and TAC were measured by colorimetric assays (TBARS Assay Kit and Antioxidant Assay Kit; Cayman Chemical Company).

### 2.9 Sample calculation

Grades of increased serum creatinine (primary endpoint) of the first 12 patients to complete the study were used for sample calculation. This was performed using the Statistical Analysis System for Windows (SAS 9.4., SAS Institute Inc, 2002‐2008, Cary, NC). This initial analysis showed that it would be necessary to include 42 patients (21 per arm).

### 2.10 Data analysis

Descriptive analyses were performed on absolute frequencies and percentages for categorical variables. Numerical variables were described by the mean and standard deviation. Statistical analyses were performed using the SAS 9.4 (SAS Institute Inc). Mann‐Whitney test was used to compare numerical variables, and Chi‐square or Fisher's exact tests were used to compare categorical variables between arms. The significance level for all analyses was 5% (*P* < 0.05).

## 3 RESULTS

### 3.1 Studied patients and characteristics

**Figure 1:**
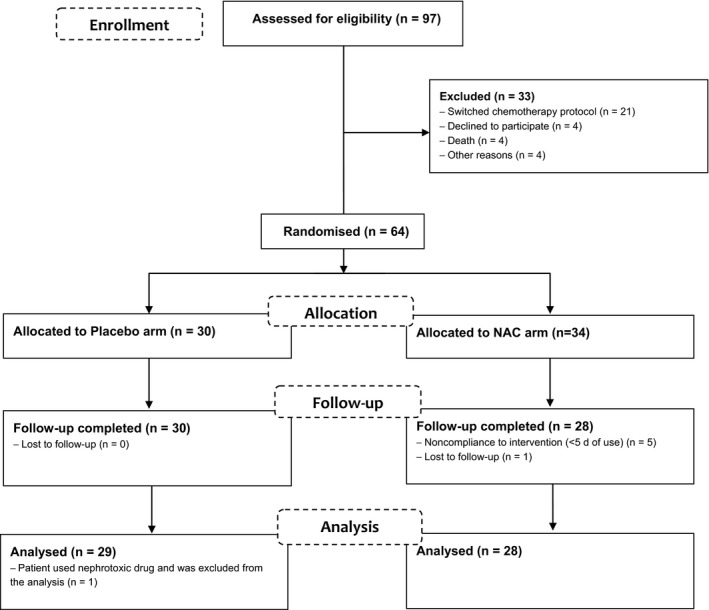
Flow diagram of the passage of participants through the trial. NAC = acetylcysteine, n = number of participants

**Figure 2:**
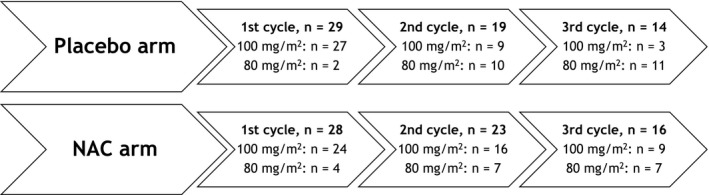
Placebo and NAC arms: number of participants, cisplatin cycles and doses. Considering the placebo arm, from 29 patients that underwent the first cycle, only 19 underwent the second cycle (19/29; 65.5%), and 14 underwent the third cycle (14/29; 48.3%). In the NAC arm, 23 from 28 patients underwent the second cycle (23/28; 82.1%) and 16 underwent the third cycle (16/28; 57.1%). In addition, 10 patients in the placebo arm (10/29; 34.5%) had their dose of cisplatin reduced after the first cycle, vs 6 in the NAC arm (6/28; 21.4%). After the second cycle, 4 patients in the placebo arm had a dose reduction (4/19; 21.1%) vs 4 in the NAC arm (4/23; 17.4%). The reasons for dose reduction or fewer cycles were toxicities and death. The NAC arm underwent more cisplatin cycles and had less dose reduction during treatment

Ninety‐seven patients provided informed consent; however, only 57 patients were analyzed (28 patients in the NAC arm vs 29 patients in the placebo arm) **(**Figure 1 **)**. In general, baseline characteristics did not differ between the 2 arms **(**Table 1 **)**. Not all patients were able to complete the 3 treatment cycles **(**Figure 2 **)**.

**Table 1:** Baseline characteristics and comparison between arms

	Placebo arm n = 29	NAC arm n = 28	*P* value
Age mean ± SD, years	56.6 ± 7.8	55.9 ± 8.8	0.8354^(a)^
BMI mean ± SD, kg/m^2^	22.6 ± 4.9	22.0 ± 3.2	0.8729^(a)^
Gender, n (%)			0.6701^(b)^
Men	27 (93.1)	25 (89.3)	
Women	2 (6.9)	3 (10.7)	
Race, n (%)			0.7297^(b)^
White	23 (79.3)	24 (85.7)	
Non‐white	6 (20.7)	4 (14.3)	
Smoking category, n (%)			0.1646^(c)^
Non‐smokers	1 (3.4)	4 (14.3)	
Light smokers	1 (3.4)	3 (10.7)	
Moderate smokers	3 (10.4)	2 (7.1)	
Heavy smokers	24 (82.8)	19 (67.9)	
Drinking category, n (%)			0.2798 ^(c)^
Abstainers	2 (6.9)	3 (10.7)	
Light drinkers	0 (0.0)	0 (0.0)	
Moderate drinkers	7 (24.1)	2 (7.1)	
Heavy drinkers	7 (24.1)	6 (21.4)	
Very heavy drinkers	13 (44.9)	17 (60.8)	
KPS, n (%)			0.5575 ^(b)^
100	2 (6.9)	4 (14.3)	
90	21 (72.4)	17 (60.7)	
80	6 (20.7)	7 (25.0)	
Tumor site, n (%)			0.9034 ^(c)^
Oropharynx	15 (51.8)	15 (53.6)	
Larynx	8 (27.6)	9 (32.2)	
Oral cavity	4 (13.8)	2 (7.1)	
Hypopharynx	1 (3.4)	2 (7.1)	
Oropharynx +oral cavity (synchronic tumors)	1 (3.4)	0 (0.0)	
Tumor differentiation, n (%)			0.7348 ^(b)^
Well differentiated	1 (3.5)	3 (10.7)	
Moderately differentiated	17 (58.6)	19 (67.9)	
Poorly differentiated	3 (10.3)	3 (10.7)	
Undifferentiated	0 (0.0)	0 (0.0)	
Not evaluated	8 (27.6)	3 (10.7)	
T stage, n (%)			0.9164 ^(c)^
T1	1 (3.5)	1 (3.6)	
T2	5 (17.2)	2 (7.1)	
T3	5 (17.2)	6 (21.4)	
T4	18 (62.1)	17 (60.8)	
Tx	0 (0.0)	2 (7.1)	
N stage, n (%)			0.3514 ^(c)^
N0	6 (20.7)	4 (14.3)	
N1	2 (6.9)	5 (17.8)	
N2	17 (58.6)	18 (64.3)	
N3	4 (13.8)	1 (3.6)	
Stage, n (%)			0.1086 ^(c)^
I	0 (0.0)	1 (3.6)	
II	1 (3.4)	0 (0.0)	
III	0 (0.0)	3 (10.7)	
IV	28 (96.6)	24 (85.7)	
Comorbidities, n (%)			0.5027 ^(c)^
Systemic arterial hypertension	11 (37.9)	4 (14.3)	
Diabetes mellitus	3 (10.3)	4 (14.3)	
Arrhythmia and other cardiovascular diseases, except SAH	2 (6.9)	0 (0.0)	
Depression	2 (6.9)	0 (0.0)	
Gastroesophageal reflux disease	1 (3.4)	1 (3.6)	
Hypothyroidism	1 (3.4)	1 (3.6)	
Arthrosis	1 (3.4)	1 (3.6)	
Other	4 (13.8)	4 (14.3)	

Note

BMI: Body Mass Index, KPS: Karnofsky Performance Status, n: absolute number, SAH: Systemic arterial hypertension, SD: standard deviation. ^(a)^Mann‐Whitney test, ^(b)^Fisher's exact test, ^(c)^Chi‐square test.

Groups were formed to perform the statistical tests for the following categories: smoking = heavy smokers vs moderate + light + non‐smokers; drinking = very heavy + heavy drinkers vs moderate + light drinkers + abstainers; tumor site = oropharynx vs other sites; tumor differentiation = moderately differentiated vs well + poorly differentiated + undifferentiated; T stage = T4 vs T1 + T2 + T3; N stage = N0 + N1 vs N2 + N3; stage = IV vs I + II + III; comorbidities = have comorbidities vs have no comorbidities.

Smoking category was classified based on the study by Jindal et al^20^. Patients were classified as non‐smokers if they reported never having smoked; light, moderate, and heavy smokers were smokers and ex‐smokers, and they were classified according to the smoking index (SI), which was the product of the average number of cigarettes smoked per day and the duration of smoking in years; light (SI = 1‐100), moderate (SI = 101‐300), and heavy (SI ≥ 301) smokers.

Drinking category was classified based on the study by Whitcomb et al^21^. Average weekly alcohol intake during the maximum lifetime drinking period (drinks/wk): abstainers, no alcohol use or <20 drinks in lifetime; light drinkers, ≤3 drinks/wk; moderate drinkers, 4‐7 drinks/wk for females and 4‐14 drinks/wk for males; heavy drinkers, 8‐34 drinks/wk for females and 15‐34 drinks/week for males; very heavy drinkers, ≥35 drinks/week.

Other comorbidities: asthma, bronchitis, chronic obstructive pulmonary disease, fibromyalgia, hepatic cirrhosis, hepatitis B, hypercholesterolemia, labyrinthitis.

### 3.2 Toxicities of treatment

Table 2 shows the toxicities of treatment and grades of severity, considering the highest grade obtained during treatment. Decreased creatinine clearance, hypomagnesemia, anemia, lymphopenia, nausea, and hearing impairment were the most frequent toxicities in both arms. When the arms were compared in relation to the grades of toxicity, there were no statistical differences, except for neutropenia where the NAC arm presented more severe neutropenia (grades 3 and 4). There was also no significant difference between arms when the grades of toxicity of serum creatinine (primary endpoint) were assessed per treatment cycle (Table 3).

**Table 2:** Grades of toxicity and comparison between arms

Toxicities/Grades	Placebo arm (%) n = 29					NAC arm (%) n = 28					*P* values
	0	1	2	3	4	0	1	2	3	4	
Increased serum creatinine	44.8	20.7	13.8	20.7	0.0	35.7	25.0	21.4	17.9	0.0	0.6517 ^(†) (a)^
Decreased creatinine clearance*	3.5	31.0	44.8	17.2	3.5	3.6	28.6	53.5	10.7	3.6	0.8620 ^(‡) (a)^
Hyperuricemia	72.4	27.6	0.0	0.0	0.0	75.0	14.3	0.0	0.0	10.7	0.8246 ^(†) (b)^
Hyponatremia	31.0	51.7	—	10.4	6.9	32.1	28.6	—	28.6	10.7	0.1128 ^(†) (a)^
Hypomagnesemia	13.8	75.9	10.3	0.0	0.0	10.7	67.9	14.3	7.1	0.0	1.0000 ^(‡) (b)^
Hypokalemia	96.6	3.4	0.0	0.0	0.0	89.3	10.7	0.0	0.0	0.0	0.3525 ^(‡) (b)^
Hypophosphatemia	75.9	—	24.1	0.0	0.0	89.3	—	10.7	0.0	0.0	0.2973 ^(‡) (b)^
Hypocalcemia	48.3	41.4	10.3	0.0	0.0	60.7	32.1	7.1	0.0	0.0	0.3459 ^(†) (b)^
Hearing impaired	24.0	32.0	28.0	16.0	0.0	20.8	16.7	25.0	37.5	0.0	0.3333 ^(†) (c)^
Hypoalbuminemia	62.1	17.2	20.7	0.0	0.0	64.3	17.8	14.3	3.6	0.0	0.8623 ^(†) (b)^
Increased AST	96.6	3.4	0.0	0.0	0.0	89.3	10.7	0.0	0.0	0.0	0.3525 ^(‡) (b)^
Increased ALT	69.0	31.0	0.0	0.0	0.0	75.0	21.4	3.6	0.0	0.0	0.6122 ^(†) (b)^
Increased ALP	89.7	10.3	0.0	0.0	0.0	89.3	10.7	0.0	0.0	0.0	1.0000 ^(‡) (b)^
Increased total bilirubin	93.2	3.4	0.0	0.0	0.0	96.4	3.6	0.0	0.0	0.0	1.0000 ^(‡) (b)^
Anemia	6.9	34.5	41.4	17.2	0.0	7.1	50.0	39.3	3.6	0.0	0.2832 ^(‡) (a)^
Leukopenia	24.1	17.3	44.8	13.8	0.0	28.6	21.4	25.0	21.4	3.6	0.4329 ^(†) (a)^
Neutropenia	44.8	20.7	27.6	6.9	0.0	42.8	14.3	10.7	28.6	3.6	0.0332 ^(†) (a)^
Lymphopenia	6.9	0.0	20.7	62.1	10.3	3.6	3.6	14.3	71.4	7.1	1.0000 ^(‡) (a)^
Thrombocytopenia	65.5	34.5	0.0	0.0	0.0	53.6	46.4	0.0	0.0	0.0	0.3581 ^(†) (b)^
Nausea	24.1	17.2	27.6	31.1	—	14.2	25.0	42.9	17.9	—	0.2155 ^(†) (a)^
Vomiting	41.4	24.1	13.8	20.7	0.0	50.0	14.2	17.9	17.9	0.0	0.8076 ^(†) (a)^

Note

^(†) ^Chi‐square test. ^(‡) ^Fisher's exact test. ^(a) ^Grade 0 vs grades 1 + 2 vs grades 3 + 4. ^(b) ^Grade 0 vs grades 1 + 2 + 3 + 4. ^(c) ^Grade 0 vs grade 1 vs grade 2 vs grade 3. — there is no grade for this toxicity. n: absolute number of patients. NAC: acetylcysteine, AST: aspartate aminotransferase. ALT: alanine aminotransferase. ALP: alkaline phosphatase. * Cockcroft–Gault formula.

**Table 3:** Grades of toxicity of increased serum creatinine after each cisplatin cycle and comparison between arms

Cycle/grades	Placebo arm (%)	NAC arm (%)	*P* value
First cycle	n = 29	n = 28	0.6484^(‡)(a)^
0	55.2	42.9	
1	20.7	35.7	
2	13.8	10.7	
3	10.3	10.7	
4	0.0	0.0	
Second cycle	n = 18	n = 23	0.9507^(†)(b)^
0	55.6	56.5	
1	33.3	26.1	
2	0.0	8.7	
3	11.1	8.7	
4	0.0	0.0	
Third cycle	n = 14	n = 16	1.0000^(‡)(b)^
0	85.7	81.3	
1	14.3	6.2	
2	0.0	12.5	
3	0.0	0.0	
4	0.0	0.0	

Note

^(†) ^Chi‐square test. ^(‡) ^Fisher's exact test. ^(a) ^Grade 0 vs grades 1 + 2 vs grades 3 + 4. ^(b) ^Grade 0 vs grades 1 + 2 + 3 + 4. n: absolute number of patients, NAC: acetylcysteine.

### 3.3 Clinical response

Clinical responses were evaluated in 25 patients of the placebo arm and in 24 patients of the NAC arm (4 patients in the NAC arm died before the exam; 3 patients in the placebo arm died; and in 1 case it was not possible to classify the clinical response). The 7 cases of acute death occurred due to cancer complications. The majority of patients had partial responses (72.0% in the placebo arm and 62.5% in the NAC arm), followed by complete response (24.0% in the placebo arm and 25.0% in the NAC arm) and progressive disease (4.0% in the placebo arm and 12.5% in the NAC arm). No patient was classified as having stable disease. Comparison of the clinical responses between the arms showed no statistical difference (*P* value = 0.9351, Chi‐square test, complete response vs partial response + progressive disease).

### 3.4 Cellular and plasmatic oxidative stress

For these analyses, only the baseline data and data obtained after the first cycle (post 5 and 20 days) are presented (Table 4). The placebo and NAC arms did not show any significant difference in the values of cellular and plasma oxidative stress biomarkers at any time point (including in the other treatment cycles; the data are not presented here because the number of samples was reduced).

**Table 4:** Cellular and plasmatic oxidative stress biomarkers and comparison between arms

	Placebo arm (n = 29)		NAC arm (n = 28)		*P* value
	n	Mean ± SD	n	Mean ± SD	
O_2_^•‐^, MFI					
Baseline	27	398.0 ± 508.4	27	293.8 ± 263.9	0.7952
5 days	23	286.8 ± 283.5	26	287.4 ± 250.1	0.3214
20 days	23	240.4 ± 237.7	21	286.9 ± 411.4	0.7960
H_2_O_2_, nmol H_2_O_2_/s (×10^‐14^)					
Baseline	28	2.8 ± 2.2	28	2.1 ± 2.0	0.2041
5 days	26	2.8 ± 2.3	27	2.3 ± 1.5	0.6889
20 days	25	3.2 ± 6.6	24	2.3 ± 1.7	0.1971
MDA, µM					
Baseline	26	9.1 ± 5.4	28	11.7 ± 6.6	0.1191
5 days	26	14.9 ± 8.1	28	13.9 ± 5.8	0.2989
20 days	24	12.6 ± 5.6	25	13.2 ± 7.3	0.2420
8‐Isoprostane, pg/mL					
Baseline	24	41.9 ± 34.1	26	62.2 ± 52.5	0.0875
5 days	24	49.8 ± 28.6	26	62.0 ± 48.2	0.1772
20 days	20	63.3 ± 93.9	21	39.0 ± 41.1	0.1087
TAC, mM					
Baseline	22	1.4 ± 0.4	26	1.4 ± 0.5	0.9670
5 days	22	1.5 ± 0.5	26	1.6 ± 0.6	0.7250
20 days	22	1.3 ± 0.4	23	1.4 ± 0.5	0.2708

Note

Mann‐Whitney test. The comparison between the arms for 5 and 20 days was based on variation from baseline.

MDA: malondialdehyde, MFI: mean fluorescence intensity, n: absolute number of patients evaluated, NAC: acetylcysteine, SD: standard deviation, TAC: total antioxidant capacity.

## 4 DISCUSSION

In this study, we investigated whether NAC could attenuate the cisplatin‐induced toxicities and oxidative stress in patients with head and neck cancer without impairing the response to treatment. Although preclinical data suggested a protective effect of NAC and other antioxidants, in our study, NAC did not limit toxicities and oxidative stress due to cisplatin.

Nephrotoxicity has been the most studied adverse effect of cisplatin and is also commonly studied to test the ability of antioxidants to attenuate cisplatin‐induced toxicities. In particular, NAC can inhibit oxidative stress, and consequently, the cisplatin‐activated signaling cascade (MAPK p58, caspase‐3, and NF‐κB) that leads to renal cell apoptosis.^26^ As observed by Shalby et al,^27^ NAC can also attenuate nephrotoxicity through its anti‐inflammatory properties, since it reduces the production of pro‐inflammatory cytokines^26^, ^28^, ^29^ and inhibits the activity of neutrophils and NF‐κB that mediate the cascade of inflammation.^30^

Abdel‐Wahab et al^14^ observed beneficial effects of NAC and taurine against cisplatin‐induced nephrotoxicity. Rats received NAC and/or taurine intraperitoneally after 3 days of cisplatin administration 3 times per week for 4 consecutive weeks. Cisplatin caused nephrotoxicity and oxidative stress, and NAC (with or without taurine) significantly improved the renal function of cisplatin‐treated rats and attenuated oxidative stress in renal tissue (MDA and TAC tests). Similar results were also obtained by Shalby et al.^27^

Unlike reports in the literature, we did not observe the protective effect of NAC against nephrotoxicity. In previous studies, high doses of NAC were used intraperitoneally in animal models. In our study, considering an average weight of 55‐60 kg and an oral dose of 600 mg/d of NAC for 7 days, approximately 10 mg/kg/d of NAC was administered to the patients, totaling 70 mg/kg. Studies that showed an important effect of NAC on nephrotoxicity used doses of 50 mg/kg/d (total 600 mg/kg),^14^ 50 mg/kg/d (total of 1200 mg/kg),^27^ 500 mg/kg/d (total of 2000 mg/kg),^31^ and 500 mg/kg/d (total of 4500 mg/kg)^32^; therefore, our dose corresponds to 2%‐20% of the daily dose and 2%‐12% of the total doses reported in these studies. Moreover, the oral bioavailability of the intact NAC ​​molecule is low, from 4% to 10%, owing to its binding to plasma proteins and the deacetylation that it undergoes in the intestinal mucosa and lumen.^33^, ^34^, ^35^, ^36^ One study showed that the levels of protection against cisplatin‐induced nephrotoxicity depend on the route of administration of NAC, and only the intravenous route was effective.^37^

Previous studies have also shown that NAC was effective in attenuating cisplatin‐induced ototoxicity^15^, ^38^ and hepatotoxicity,^16^ in contrast to the current study, probably owing to the different doses, schedules, and routes chosen. No study using NAC against myelotoxicity, nausea, and vomiting induced by cisplatin was found; however, research using other antioxidants showed beneficial results.^7^, ^39^, ^40^, ^41^, ^42^ In this study, we also quantified ROS in PBMCs, lipid peroxidation, and TAC in the plasma as markers of oxidative stress. Our results showed that the NAC and placebo arms did not differ in these analyses.

It is known that antioxidants can increase cell proliferation in tumors^43^ or impair the effect of antineoplastic drugs.^17^ However, studies have shown no evidence that antioxidants were associated with decreased survival or tumor responses.^44^ In the case of NAC, as it is a precursor of glutathione which participates in the mechanism of cisplatin inactivation, the effect of cisplatin could be reduced. In addition, NAC may inhibit the pathway by which cisplatin induces apoptosis (caspases), impairing its cytotoxic action.^45^ Muldoon et al,^18^ using a tumor model, showed that when NAC ​​is administered 30 min before cisplatin administration, a reduction in the action of cisplatin may occur, but not when it is administered 4 h after. According to Dickey et al,^37^ the reduction in the antineoplastic effect of cisplatin could be avoided by separating cisplatin and NAC administration spatiotemporally. Robbins et al^46^ determined that this space separation occurs owing to the administration of cisplatin and NAC through different routes. In our study, NAC administration and cisplatin administration were spatiotemporally separated, and there was no difference in clinical response between the patients receiving NAC and placebo.

The antioxidant type, dose, route, and regimen of administration used in our study were based on a few factors. Among the several compounds that have been studied in an attempt to prevent the toxicities of cisplatin, such as some vitamins and minerals, taurine, dimethylurea, d‐methionine, and linseed oil, NAC ​​was chosen for this study since animal model studies showed promising results of NAC ​​in the attenuation of cisplatin toxicities. NAC is also a drug approved in Brazil and easy to access (low cost), safe, and does not present frequent adverse effects, and therefore, was considered to be a good candidate to be included as a support medication during treatment with cisplatin. The choice of dosage form (syrup) and route of administration (oral) were made, because oral use is noninvasive and the placebo was easier to prepare. Intravenous administration of NAC ​​for 7 days would have been difficult because the patients studied were not hospitalized. In relation to the dose, we opted to supplement patients at a low‐dose to not interfere with antitumor efficacy, since the doses used in animal models were extremely high, and the effect on tumor progression has not yet been fully elucidated in the literature. The chosen dose has already been used in another study^38^ and is approved by the Brazilian Health Regulatory Agency (Anvisa) for the treatment of productive cough. The syrup was given once a day at night to facilitate patient adherence. Thus, there was an interval between the administration of cisplatin and NAC (~12 h). NAC was administered 2 days before chemotherapy as a prophylaxis and on the subsequent 5 days, when the main toxicities, such as nausea, vomiting, nephrotoxicity, and oxidative stress, were likely to occur.

The limitations of our study included difficulties with blood sample collection for oxidative stress assays; at one time, some patients were so debilitated that the health professional had difficulty accessing their veins. We performed analysis during all 3 cycles of chemotherapy; however, the oxidative stress analyses were impaired after the second cycle of treatment owing to loss of samples due to these reasons. Regarding cellular oxidative stress assays, the large variances when performing statistical analyses may be a consequence of the complexity of the assays, in which readings can be influenced by many factors, such as minor differences in pH, temperature, and air bubbles in the reaction chamber and plate. It is our pleasure to recommend a few suggestions for further trials in this area: include a greater number of participants; test other doses, schedules, and routes of administration of NAC; perform the second radiological assessment 8 or 12 weeks after completion of treatment to decrease the false‐positives related to local inflammation and fibrosis induced by chemoradiation; and determine late deaths and overall survival in both arms.

Although this study has shown that the use of NAC was not effective in attenuating the toxicity of cisplatin in patients with head and neck cancer, this was the first double‐blind, placebo‐controlled, randomized trial using this approach, and should form a basis to conduct new studies in humans. It is a challenge for future clinical studies to identify an optimal dose and regimen for NAC delivery to protect against cisplatin toxicities without compromising its antineoplastic efficacy during clinical treatment.

## 5 CONCLUSION

Low‐dose oral NAC does not protect patients with head and neck cancer from cisplatin‐induced toxicities and oxidative stress. The antitumor efficacy of cisplatin was apparently not impaired by NAC. Further clinical studies should be conducted to test other doses, schedules, and routes of administration of NAC.

## CONFLICT OF INTEREST STATEMENT

The authors declare that they have no conflicts of interest to disclose.
